# A Case of Severe, Difficult-to-Diagnose Legionnaires' Disease in a Young Welder

**DOI:** 10.7759/cureus.42250

**Published:** 2023-07-21

**Authors:** Dylan B McBee, Ruth Mizu, Ahmed M Hamdi

**Affiliations:** 1 Department of Medicine, Section of Infectious Diseases, Baylor College of Medicine, Houston, USA

**Keywords:** legionella pneumophila, diagnostic errors, mechanical ventilation, welder, legionella pneumonia

## Abstract

Legionellosis among welders and other metalworkers is a rare but potentially underappreciated occupational hazard. The same mechanisms that predispose welders to severe pneumonia from *Streptococcus pneumoniae* and *Bacillus cereus* may similarly predispose them to *Legionella pneumophila *infection. We present a case of a previously healthy, immunocompetent 31-year-old male welder presenting with three days of shortness of breath, hypoxia, high-grade fever, and blood-tinged sputum. Chest computed tomography (CT) revealed a lobar consolidation of the right middle and lower lobes. Laboratory evaluation showed borderline hyponatremia, hypophosphatemia, and elevated liver enzymes. The patient was ultimately intubated and started on broad-spectrum antibiotics. Multiple respiratory cultures were negative and *Legionella* urine antigen testing was also negative. Eventually, bronchial *Legionella *culture was positive for *Legionella pneumophila, *and a blood next-generation sequencing test also confirmed the diagnosis. He was extubated six days following admission and subsequently discharged.

## Introduction

Legionellosis broadly describes any human disease caused by *Legionella*, a genus of intracellular, Gram-negative bacteria [1]. Infection in humans is classically characterized by either pneumonic (Legionnaires’ disease) or non-pneumonic (Pontiac Fever) manifestations [1]. *Legionella pneumophilia* serogroup 1 serves as the leading cause of legionellosis in the United States with other serogroups and species contributing less significantly to overall infection rates [2]. Legionellosis is estimated to contribute to 2-10% of community-acquired pneumonia (CAP) and disproportionately contributes to CAP requiring inpatient and more critical care [3,4]. Despite estimations that legionellosis is both underdiagnosed and underreported,­­ the incidence of this disease has grown significantly and should be considered an increasing threat to public health [2].

In this case report we highlight the growing domain of *Legionella pneumophila* by depicting an extreme case of Legionnaires' disease in a relatively healthy 31-year-old male welder. We reflect on this underappreciated occupational threat with reference to pneumococcus and anthrax toxin-producing *Bacillus cereus* as contributors to occupational pneumonia among metalworkers. Our case also serves to emphasize the diagnostic difficulties of legionellosis while showcasing the consequences of delayed and inadequate antibiotic therapy for *Legionella* pneumonia.

## Case presentation

A 31-year-old African American male welder with no significant past medical history and a one-pack-per-day smoking history of unknown duration presented to an outside medical facility following three days of progressive shortness of breath and cough with delayed-onset blood-streaked sputum. A subjective fever and generalized body aches of the same duration accompanied the patient’s pulmonary symptoms, but he denied vomiting, diarrhea, chest pain, sore throat, rashes, and any sick contacts. The patient has worked as a ship welder for three years on the Gulf Coast traveling between southeast Texas and Louisiana for work. His job requires working irregular hours, typically 10- to 12-hour shifts in a poorly ventilated room. Despite using a respirator at work, he routinely inhales metal fumes. However, he had never endorsed hemoptysis or such significant shortness of breath in the past.

At an outside hospital, the patient was found to be febrile to 104.7 °F and tachypneic with an oxygen saturation in the mid-to-high 80s. A chest radiograph demonstrated a right lower lobe consolidation concerning for pneumonia and he was started on intravenous vancomycin and piperacillin-tazobactam. He initially required to 5-6 L of oxygen via nasal cannula while maintaining an oxygen saturation in the low 90s. Despite these interventions, the patient continued to have worsening respiratory distress. It was at this point that the patient was transferred to our institution for more acute management and further workup of his pneumonia.

**Figure 1:**
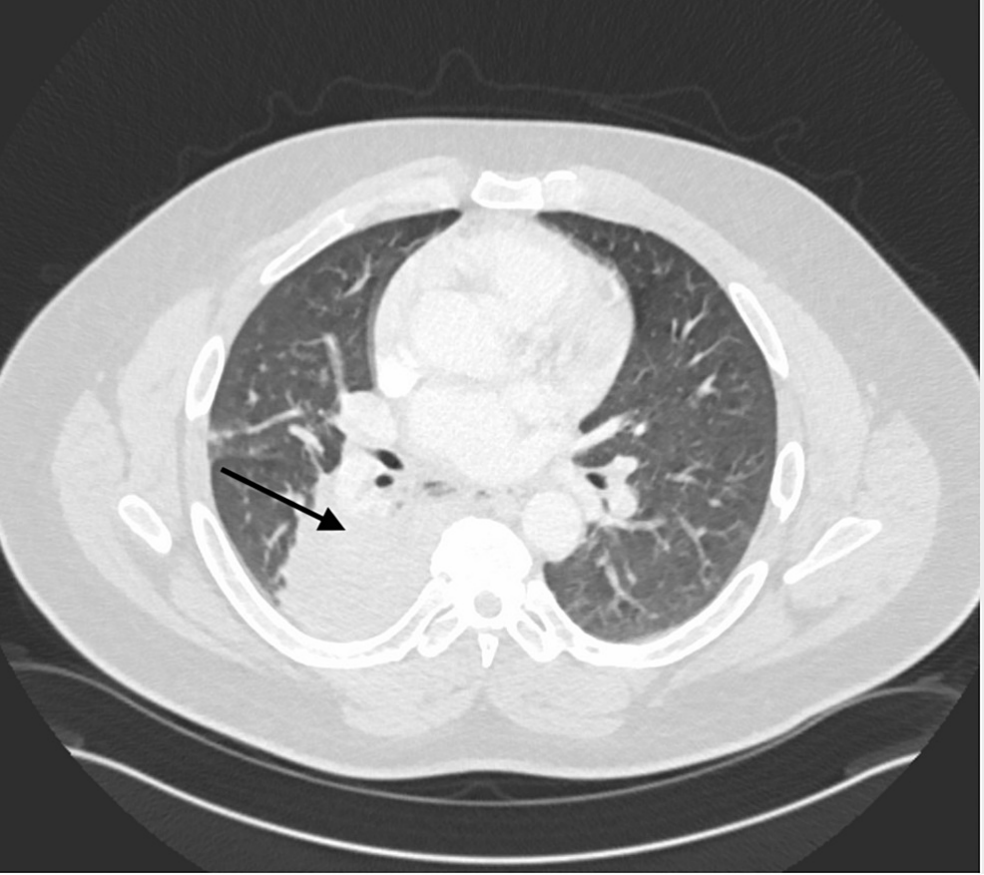
Chest CT demonstrating airspace consolidation of the right middle and lower lobe suggestive of pneumonia CT: computed tomography

Upon presentation, the patient was lethargic with labored breathing and a respiratory rate in the mid-40s but was otherwise hemodynamically stable. A physical exam demonstrated coarse breath sounds bilaterally and a chest computed tomography (CT) scan (Figure 1) confirmed a right middle and lower lobe consolidation suggestive of pneumonia. In addition, imaging revealed superimposed ground-glass opacities in bilateral lower lobes. The patient was admitted to the intensive care unit for acute hypoxemic respiratory failure and initially trialed on bilevel positive airway pressure (BiPAP) but was eventually intubated for increased work of breathing.

Laboratory evaluation revealed a complete blood count significant for leukocytes of 13.8 (4-10 x 10^9^/L) with neutrophilic predominance. An elevated procalcitonin of 0.45 (< 0.05 ng/mL) was also noted but the patient’s D-dimer and venous lactic acid were within normal limits. Serum chemistry revealed a mildly lowered serum sodium of 134 meq/L (136-145 meq/L) and serum phosphate of 1.7 mg/dL (2.3-4.7 mg/dL). Liver enzymes were marginally elevated on admission and continued to rise until an aspartate aminotransferase (AST) of 214 (8-48 U/L) and alanine aminotransferase (ALT) of 198 (7-55 U/L) were reported on day five.

Initial microbiological testing was largely unrevealing. Rapid Influenza A&B screening, SARS-CoV-2 reverse transcription-polymerase chain reaction (RT-PCR), and a comprehensive respiratory panel all resulted in negative. Additionally, pneumococcal and *Legionella* urinary antigen tests (UATs) were both negative. Blood cultures were drawn but no growth was observed. An upper respiratory tract sputum culture was not taken. Instead, bronchoalveolar lavage was performed with 1+ white blood cells and a gram stain showing less than 1+ gram-negative rods but no growth on cultures. Acid-fast bacteria smear and culture and fungus smear and culture were also negative. Chlamydia PCR and Mycoplasma PCR were both negative. Finally, a specialized *Legionella* culture utilizing the bronchoalveolar lavage sample came back positive for *Legionella pneumophila* five days following bronchoscopy. In addition, a next-generation sequencing (NGS) of plasma microbial cell-free DNA (mcfDNA) test that was ordered earlier also confirmed the presence of *Legionella pneumophila*.

Upon presenting to our hospital, the patient was trialed on a broad-spectrum regimen of intravenous vancomycin, ciprofloxacin, and meropenem. However, with consideration for anthrax toxin-expressing *Bacillus cereus*, vancomycin was discontinued in favor of linezolid because of its ability to suppress bacterial toxin production [5]. Doxycycline was added to the existing regimen on day two of admission but was subsequently discontinued on day five due to concern for rising liver enzymes. When the diagnosis was confirmed, meropenem was discontinued. Later, ciprofloxacin was transitioned to levofloxacin upon discharge.

The patient’s acute hypoxic respiratory failure improved with antibiotic therapy, and he was extubated six days following admission. The patient has not experienced any complications requiring readmission and is now one year out from discharge.

## Discussion

Legionellosis may occur following inhalation or aspiration of droplets containing the pathogen [1]. *Legionella pneumophila* can contaminate human-made water systems that then aerosolize the pathogen priming it for human inoculation [1]. The occupational risk of legionellosis is highly variable with outbreaks routinely spanning nearly all workplace settings [6]. Despite this, industrial and manufacturing sites report the highest incidence of *Legionella* outbreaks among workplaces [6].

Together, several laboratory and clinical abnormalities may be used to guide early presumptive clinical suspicion for *Legionella* [7]. Despite many overlapping clinical characteristics with other types of pneumonia including fever, cough, and shortness of breath, legionellosis may be in part distinguished by gastrointestinal symptoms such as nausea, vomiting, or diarrhea [7]. While not specific, *Legionella* is resistant to monotherapy with beta-lactam antibiotics and should be considered with the failure of CAP to respond to treatment [8]. Clinical suspicion may also be supported by laboratory abnormalities including hyponatremia, hypophosphatemia, elevated C-reactive protein levels, or elevated hepatic transaminases [7,9]. Interestingly, our patient did not endorse any gastrointestinal symptoms. Additionally, there was some concern for doxycycline-induced acute liver toxicity resulting in transaminitis however this was more likely a signature of his primary disease [10]. While a combination of clinical signs and symptoms may increase the index of suspicion, all patients with moderate to severe CAP should be tested, and empirically treated, for *Legionella* [11].

Given the significant morbidity and mortality that may be associated with legionellosis, clinicians should maintain a high index of suspicion even in the face of diagnostic uncertainty to ensure appropriate antibiotic therapy or further delay the appropriate escalation of care [7]. As seen with our patient, a negative *Legionella* UAT should not preclude this diagnosis. *Legionella* UATs provide greater clinical value when ruling in disease by providing high specificity [12]. However, this is only true for legionellosis caused by the most common causal species, *Legionella pneumophila* serotype 1 for which this test was designed [12]. A multicenter, prospective study of 1941 adults hospitalized with CAP demonstrated a mere specificity of 35% (95% CI 33-37%) and only 63% sensitivity (95% CI 44-79%) using the* Legionella pneumophila* UAT [7]. Hence, a specialized *Legionella* culture of lower respiratory tract samples should be considered in high-suspicion cases to exclude this diagnosis [12]. Cultures taken from the upper respiratory tract alone may not suffice [12]. Notably,* Legionella* species are nutritionally fastidious and would not grow on regular culture plates. Instead, the specimen must be incubated in *Legionella-*specialized cultures containing buffered charcoal yeast extract (BCYE) plates with L-cysteine [1].

Excessive inhalation of metal fumes, and in particular iron, contributes significantly to the risk and severity of pulmonary disease [13-16]. Metalworkers are known to demonstrate higher rates of pneumonia compared to other industrial workers [13,14,17]. Additionally, more than five decades of work now clearly demonstrates a higher case-fatality rate among metalworkers who develop pneumonia [14]. In fact, the risk of death from pneumococcal pneumonia appears to be particularly elevated [13,14]. Somewhat surprisingly, excessive mortality may be limited to metalworkers below the standard retirement age of 65 and thus those with ongoing exposure to metal fumes [18]. Similarly, rates of pneumonia are higher in welders compared to other workers, but only when exposure to metal fumes has occurred within the previous year (OR = 1.6; CI = 1.1-2.4) [17]. Taken together, occupational exposure to metal fumes may acutely enhance workers’ susceptibility to severe manifestations of pneumonia.

Given the diagnostic challenges of this case, a rare occupation-related pneumonia unique to welders and other metalworkers was briefly considered in our patient. Welder’s anthrax refers to an infection caused by an anthrax toxin-expressing species of *Bacillus cereus *that presents similarly to inhalation anthrax [15]. Among the few cases previously described, each closely resembles the presentation of our patient. While only nine such cases were reported in the United States between 1997 and 2023, all persons were metalworkers who had worked in southeast Texas or Louisiana [15]. All nine patients were immunocompetent men of working age, yet all developed severe dyspnea and hemoptysis [15]. Like our patient, many of these men also had a positive smoking history [15].

Ultimately, metalworkers’ predisposition to severe pneumonia may be explained by both acute and chronic pathologic changes to the respiratory system. Acutely, exogenous iron serves as a nutrient for microorganisms promoting growth [13,15]. Inhaled particulate matter residing in the airway system may also enhance adherence to CAP pathogens while promoting free radical injury of innate host defenses [13,15]. Prolonged exposure to inhaled metals in welders is associated with fibrotic changes that manifest as respiratory bronchiolitis initially and with more significant exposure, may develop into desquamative interstitial pneumonitis [19]. In a patient with a positive smoking history, this process is likely to be exacerbated.

To our knowledge, legionellosis has not been extensively linked to welders or other metalworkers. In 2005, a contaminated spot-welding water bath resulted in two welders falling ill with *Legionella* with both requiring inpatient hospital care [20]. The authors concluded that industrial spot-welding baths pose a significant and previously under-recognized source of infection [20]. To date, no further outbreaks among welders have been reported. The relative paucity of work linking occupation-related *Legionella* with welders would suggest that this threat may be far underappreciated. An extensive body of work already demonstrates increased susceptibility to both acute and chronic pulmonary disease in welders. It is likely that the pathologic mechanisms that increase the risk of pneumococcal and *Bacillus cereus* pneumonia in welders may also contribute to increased incidence and severity of legionellosis. Further, the industrial occupational environment of welders portends favorably to *Legionella* inoculation in a space dense with metal fumes and aerosolized water droplets. Ultimately, this case report serves as a reminder that *Legionella pneumophila* should be considered among the differential diagnoses for welders and other metalworkers with pneumonia. In addition, underlying occupation-related pulmonary dysfunction may predispose these individuals to more critical diseases requiring a higher level of care.

## Conclusions

*Legionella pneumophila* is an underreported but increasingly common cause of CAP globally that often requires inpatient and more critical care. Welders and other workers exposed to metal fumes are at increased risk of contracting severe, life-threatening pneumonia from several pathogens. Both acute and chronic pathologic changes leading to respiratory dysfunction contribute to significant morbidity seen with *Legionella* and other bacterial causes of pneumonia. Clinical signs and symptoms of legionellosis may be varied and UATs should not be relied upon to dismiss the disease. Thus, confirming the diagnosis of *Legionella* pneumonia may be challenging and requires the use of specialized cultures or even next-generation sequencing serum tests. Ultimately, stricter adherence to industry standards, like the use of respirators, may be used to mitigate occupation-related injury.

## References

[1] Gonçalves IG, Simões LC, Simões M (2021). Legionella pneumophila. Trends Microbiol.

[2] Viasus D, Gaia V, Manzur-Barbur C, Carratalà J (2022). Legionnaires' disease: update on diagnosis and treatment. Infect Dis Ther.

[3] Sabrià M, Campins M (2003). Legionnaires' disease: update on epidemiology and management options. Am J Respir Med.

[4] Andrea L, Dicpinigaitis PV, Fazzari MJ, Kapoor S (2021). Legionella pneumonia in the ICU: a tertiary care center experience over 10 years. Crit Care Explor.

[5] Head BM, Alfa M, Sitar DS, Rubinstein E, Meyers AF (2017). In vitro evaluation of the effect of linezolid and levofloxacin on Bacillus anthracis toxin production, spore formation and cell growth. J Antimicrob Chemother.

[6] Principe L, Tomao P, Visca P (2017). Legionellosis in the occupational setting. Environ Res.

[7] Bellew S, Grijalva CG, Williams DJ (2019). Pneumococcal and legionella urinary antigen tests in community-acquired pneumonia: prospective evaluation of indications for testing. Clin Infect Dis.

[8] Sopena N, Sabrià-Leal M, Pedro-Botet ML, Padilla E, Dominguez J, Morera J, Tudela P (1998). Comparative study of the clinical presentation of Legionella pneumonia and other community-acquired pneumonias. Chest.

[9] Cunha BA (2008). Severe Legionella pneumonia: rapid presumptive clinical diagnosis with Winthrop-University Hospital's weighted point score system (modified). Heart Lung.

[10] Heaton PC, Fenwick SR, Brewer DE (2007). Association between tetracycline or doxycycline and hepatotoxicity: a population based case-control study. J Clin Pharm Ther.

[11] Metlay JP, Waterer GW, Long AC (2019). Diagnosis and treatment of adults with community-acquired pneumonia. An official clinical practice guideline of the American Thoracic Society and Infectious Diseases Society of America. Am J Respir Crit Care Med.

[12] Peci A, Winter AL, Gubbay JB (2016). Evaluation and comparison of multiple test methods, including real-time PCR, for legionella detection in clinical specimens. Front Public Health.

[13] Wong A, Marrie TJ, Garg S, Kellner JD, Tyrrell GJ (2010). Welders are at increased risk for invasive pneumococcal disease. Int J Infect Dis.

[14] Palmer KT, Cosgrove MP (2012). Vaccinating welders against pneumonia. Occup Med (Lond).

[15] de Perio MA, Hendricks KA, Dowell CH (2022). Welder's anthrax: a review of an occupational disease. Pathogens.

[16] Coggon D, Palmer KT (2016). Are welders more at risk of respiratory infections?. Thorax.

[17] Palmer K, Coggon D (1997). Does occupational exposure to iron promote infection?. Occup Environ Med.

[18] Coggon D, Inskip H, Winter P, Pannett B (1994). Lobar pneumonia: an occupational disease in welders. Lancet.

[19] Cosgrove MP (2020). Interstitial lung disease in welders. Occupational and Environmental Lung Dis.

[20] (2022). Investigation of a legionellosis outbreak in a welding workshop. https://www.health.vic.gov.au/publications/investigation-of-a-legionellosis-outbreak-in-a-welding-workshop.

